# Supplementary material for: Terahertz chiral photonic-crystal cavities for Dirac gap engineering in graphene
Source: Nat Commun. 2025 Jun 6;16:5270. doi: 10.1038/s41467-025-60335-x (PMC12144285; doi:10.1038/s41467-025-60335-x)
Supplement: Supplementary file 1 — Supplementary Information [file 41467_2025_60335_MOESM1_ESM.pdf]

Supplementary Information: Terahertz chiral photonic-crystal cavities for Dirac gap engineering in graphene

Fuyang Tay[1,2*], Stephen Sanders[1], Andrey Baydin[1,3,4], Zhigang Song[5], Davis M. Welakuh[6], Alessandro Alabastri[1,3,4], Vasil Rokaj[7,8,9], Ceren B. Dag[7,8,10], Junichiro Kono[1,3,4,11,12*]

[1]Department of Electrical and Computer Engineering, Rice University, Houston, 77005, Texas, USA.
[2]Applied Physics Graduate Program, Smalley–Curl Institute, Rice University, Houston, 77005, Texas, USA.
[3]Smalley–Curl Institute, Rice University, Houston, 77005, Texas, USA.
[4]Rice Advanced Materials Institute, Rice University, Houston, Texas 77005, USA.
[5]John A. Paulson School of Engineering and Applied Sciences, Harvard University, Cambridge, 02139, Massachusetts, USA.
[6]Max Planck Institute for the Structure and Dynamics of Matter, Luruper Chaussee 149 Hamburg, 22761, Germany.
[7]Department of Physics, Harvard University, Cambridge, 02138, Massachusetts, USA.
[8]ITAMP, Harvard-Smithsonian Center for Astrophysics, Cambridge, 02138, Massachusetts, USA.
[9]Department of Physics, Villanova University, Villanova, 19085, Pennsylvania, USA.
[10]Department of Physics, Indiana University, Bloomington, 47405, Indiana, USA.
[11]Department of Physics and Astronomy, Rice University, Houston, 77005, Texas, USA.
[12]Department of Materials Science and NanoEngineering, Rice University, Houston, 77005, Texas, USA.

*Corresponding author(s). E-mail(s): fuyang.tay@columbia.edu; kono@rice.edu;

Contents

1 Comparison with transfer matrix method calculations **2**

2 Effects of losses in PCCs **3**

3 Chiral 1D-PCCs with five magnetoplasma layers **4**

4 Magnetic Field Dependence of the Chiral PCCs **5**

5 Chiral Tamm cavities **6**

6 Calculations of vacuum electric field **7**

S1 Comparison with transfer matrix method calculations

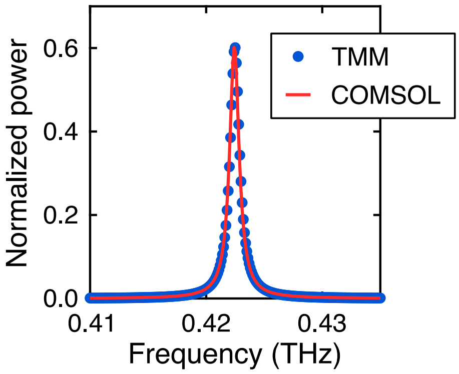

Supplementary Fig. 1 Comparison of calculations performed using TMM and COMSOL. Normalized power spectra for incident light with the "-" polarization at $B = 0.212\,\mathrm{T}$ calculated using TMM and COMSOL Multiphysics.

The calculations of the transmittance spectra and cavity mode profiles can be reproduced using the transfer matrix method (TMM). For instance, the transmitted power spectra of the chiral PCC for circularly polarized light, calculated using TMM

and COMSOL, exhibit excellent agreement, as shown in Supplementary Fig. 1. However, for linearly polarized incident light, the standard isotropic TMM is required to be extended to an anisotropic version [1] to account for the off-diagonal elements of the permittivity tensor. In contrast, COMSOL provides a more convenient approach by allowing direct incorporation of a full 3×3 permittivity tensor without additional modifications. In addition, COMSOL facilitates the exploration of more sophisticated designs in future studies, such as a chiral 1D-PCC with a metasurface array on the defect layer surface to further reduce the mode volume [2, 3].

S2 Effects of losses in PCCs

According to the figure in the main text, $\tilde{\varepsilon}_{-} \approx 12.298 - 0.104i$ at ω_{cav}, which corresponds to $\tilde{n} = 3.507 - 0.015i$.

To understand why the transmitted power and confined electric field decrease significantly when layer (iii) is InSb, we used TMM to investigate transmittance spectra of 1D-PCCs with different values of losses. Note that the TMM used the $\tilde{n} = n + i\kappa$ convention, which is opposite to the convention for the complex permittivity used in the main manuscript.

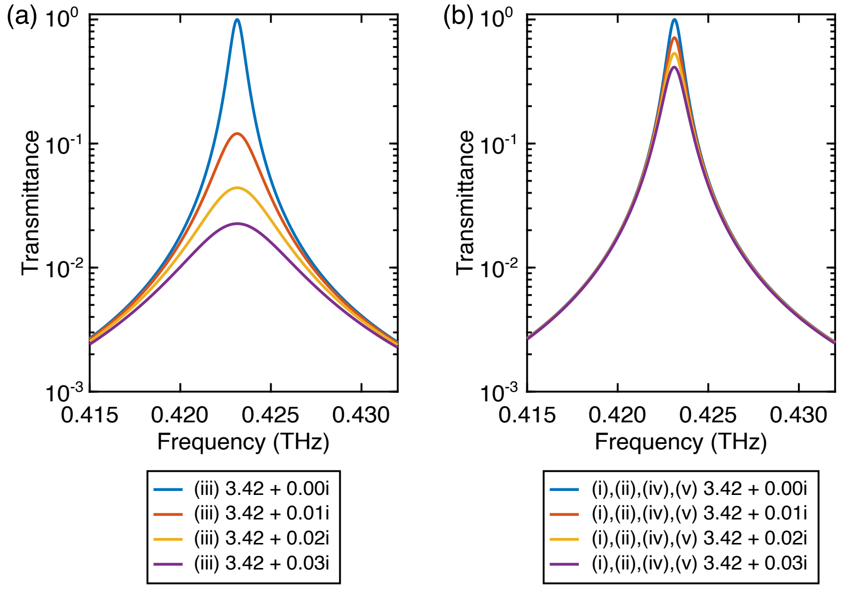

Supplementary Fig. 2 Effects of losses on transmittance spectra. The transmittance spectra when κ of (a) layer (iii) and (b) layers (i)–(ii) & (iv)–(v) increases, respectively. The spectra were calculated using the TMM.

In the calculations, $n = 3.42$ for all layers, but the value of κ increases for different layers. Both n and κ are constant as a function of frequency. Supplementary

Figure 2(a) shows the spectra when the κ of the defect layer (layer (iii)) increases, while Supplementary Fig. 2(b) shows the spectra when the κ for all other layers (layer (i)–(ii) & (iv)–(v)) increases. The calculations show that the transmitted power is more sensitive to the losses in the defect layer than the losses in other layers. Thus, the transmitted power and the confined electric field are much lower when layer (iii) is replaced with InSb.

S3 Chiral 1D-PCCs with five magneto-plasma layers

Supplementary Figure 3(a) and (b) show that the transmitted power and electric field are greatly suppressed if the chiral 1D-PCC consists of five InSb layers. Even if we reduce the thickness of the defect layer slightly to shift the peak frequency to the bare cavity mode frequency, the transmitted power and the electric field remain weak.

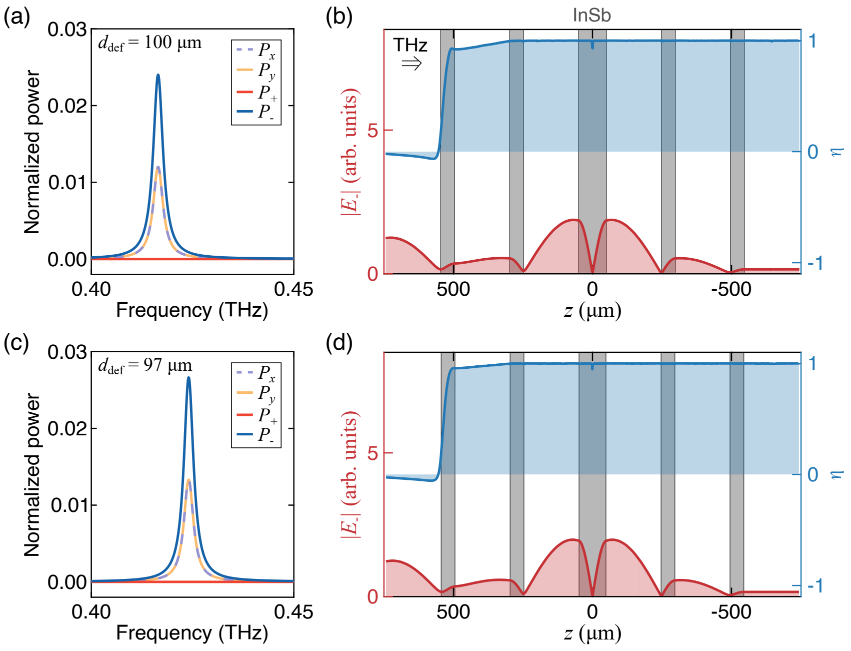

Supplementary Fig. 3 Transmittance spectra and mode profiles of a chiral 1D-PCC consisting of five InSb layers. The simulation results when the thickness of the layer (iii) is (a)–(b) 100 μm and (c)–(d) 97 μm, respectively.

S4 Magnetic Field Dependence of the Chiral PCCs

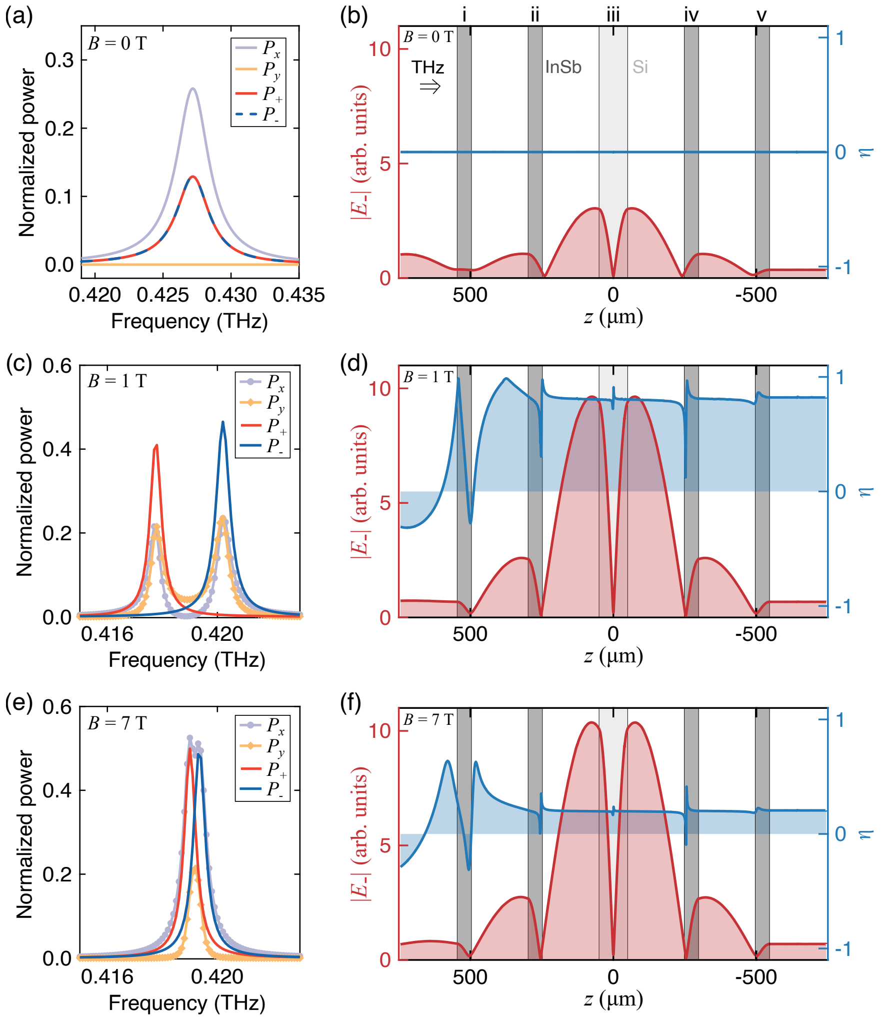

Supplementary Fig. 4 The properties of the optimized chiral 1D-PCCs (Design II) at different magnetic fields, B. Transmittance spectra and mode profiles of the chiral 1D-PCCs at (a)–(b) $B = 0\,\mathrm{T}$, (c)–(d) $B = 1\,\mathrm{T}$, and (e)–(f) $B = 7\,\mathrm{T}$, respectively. All layers are InSb except layer (iii). The $|E_-(z)|$ and $\eta(z)$ profiles are plotted at the peak frequency of P_-. The incident light is linearly polarized in the x axis.

We examined the properties of the cavity mode of our optimized chiral PCC design at three different magnetic fields ($B = 0\,\mathrm{T}$,$1\,\mathrm{T}$, and $7\,\mathrm{T}$), as shown in Supplementary

Fig. 4. The structure is Design II: layers (i)–(ii) and (iv)–(v) are InSb whereas layer (iii) is silicon. At $B = 0\,\mathrm{T}$, the PCC is linear since TRS is not broken. Consequently, the transmitted light remains linearly polarized [Supplementary Fig. 4(a)] and $\eta(z)$ is close to 0 [Supplementary Fig. 4(b)]. Note that the peak amplitude of $|E_-(z)|$ in the chiral 1D-PCC is lower than that in the linear 1D-PCC due to losses in the magnetoplasma.

At $B = 1\,\mathrm{T}$, the transmittance spectrum for P_x exhibits two peaks, as shown in Supplementary Fig. 4(c). This is because the peak frequencies differ for the two opposite handedness; the peak frequency of P_- is higher than that of P_+. This feature can be utilized as a filter for circularly polarized light. However, $\eta(z) \neq 1$ at z_{max} [Supplementary Fig. 4(d)], indicating that the cavity electric field is not fully circularly polarized at this B.

At high B, such as $B = 7\,\mathrm{T}$, ω_{c} in InSb exceeds ω_{cav} significantly. The peaks for P_+ and P_- coalesce [Supplementary Fig. 4(e)], and $\eta(z_{\mathrm{max}})$ decreases to 0 [Supplementary Fig. 4(f)]. Therefore, these simulation results show that the chiral 1D-PCC is effective only when ω_{c} and ω_{cav} are approximately equal.

The properties of the chiral PCC at $B = -0.212\,\mathrm{T}$ are identical to those at $B = 0.212\,\mathrm{T}$, except that the roles of P_- and P_+ are interchanged and $\eta(z = z_{\mathrm{max}}) = -1$, as shown in Supplementary Fig. 5.

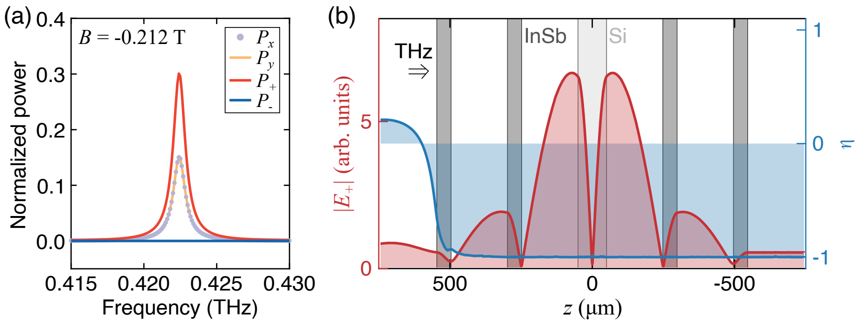

Supplementary Fig. 5 The properties of the chiral 1D-PCC at negative B. (a) Transmittance spectrum and (b) mode profile of the chiral cavity mode at $B = -0.212\,\mathrm{T}$. The incident light is linearly polarized in the x direction.

S5 Chiral Tamm cavities

This section studies chiral Tamm cavities. The Tamm cavities consist of three silicon layers with identical thicknesses separated by air. The thicknesses of the silicon and air layers are 50 μm and 198 μm, respectively. The back side of the third silicon layer is coated by a 100 nm gold (Au) layer.

Supplementary Figure 6(a) and (b) display reflected power spectra and mode profiles of the Tamm cavity. The incident light is linearly polarized in the x direction. The reflected power spectra show a resonance at 0.4225 THz, and the reflected light

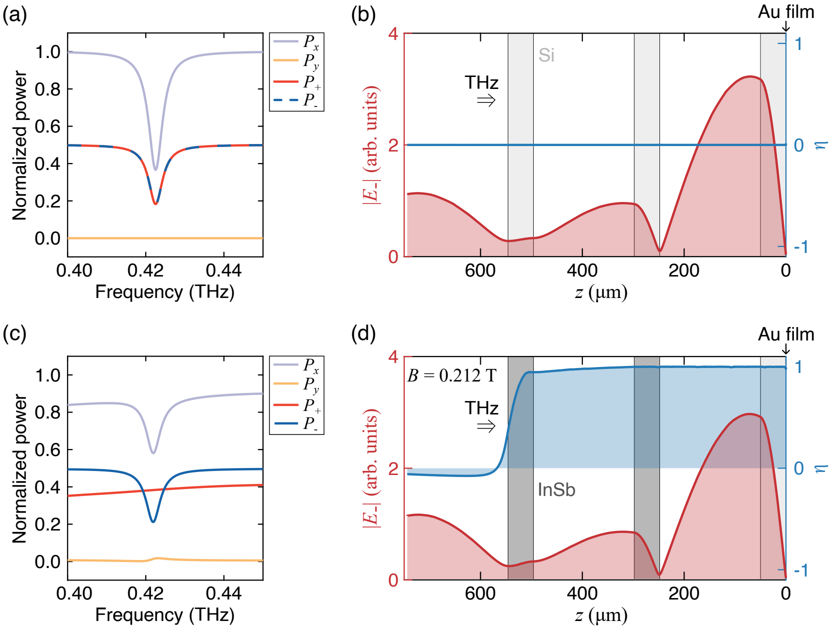

Supplementary Fig. 6 Transmittance spectra and mode profiles of chiral Tamm cavities. The simulation results for (a)–(b) a linear Tamm cavity and (c)–(d) a chiral Tamm cavity.

is linearly polarized ($P_x \approx 2P_+ = 2P_-, P_y \approx 0$). The electric field is mainly localized at the front side of the third silicon layer.

By replacing the first two silicon layers with InSb, the resonance of the Tamm cavity becomes chiral. The reflected power spectra and mode profiles of a chiral Tamm cavity are shown in Supplementary Fig. 6(c) and (d). An external magnetic field of $B = 0.212\,\mathrm{T}$ is considered in the calculation. For the chiral Tamm cavity, the reflected power $P_+ > 0$ because the front side of the first wafer reflects light in both circular polarizations. However, the cavity resonance is only observable in the P_- spectrum. The mode profile indicates that the electric field at the maximum position is circularly polarized ($\eta \approx 1$).

S6 Calculations of vacuum electric field

The mode profiles of the chiral cavity modes were calculated using COMSOL Multiphysics software. In this case, we assumed that the cavity was excited by a circularly polarized plane wave with an electric field amplitude of $1\,\mathrm{V/m}$. To calculate the variance of the electric field in the vacuum state, $E_{\text{vac,-}}$, we normalized the electric field extracted from the simulations, $E_{\text{sim,-}}$, such that the following relation is satisfied [4, 5]:

$$\int_V dr \varepsilon_0 \varepsilon_r(r) E^2_{\text{vac,-}}(r) = \frac{\hbar\omega}{2}, \tag{S1}$$

where ε_r denotes the relative permittivity, $\hbar$ denotes the reduced Planck's constant, and V denotes the cavity volume.

$E_{\text{vac,-}}$ can be expressed as

$$E_{\text{vac,-}}(z) = \sqrt{\frac{\hbar\omega}{2\varepsilon_0 S}} \frac{E_{\text{sim,-}}(z)}{\sqrt{\int_{L_z} dz \varepsilon_r(z) E^2_{\text{sim,-}}(z)}}. \quad \text{(S2)}$$

S is the surface area of the cavity and L_z is the effective cavity length spanning from the front side of the first layer to the back side of the last layer.

The vacuum field profiles of the chiral 1D-PCC and the chiral Tamm cavity are shown in Supplementary Fig. 7.

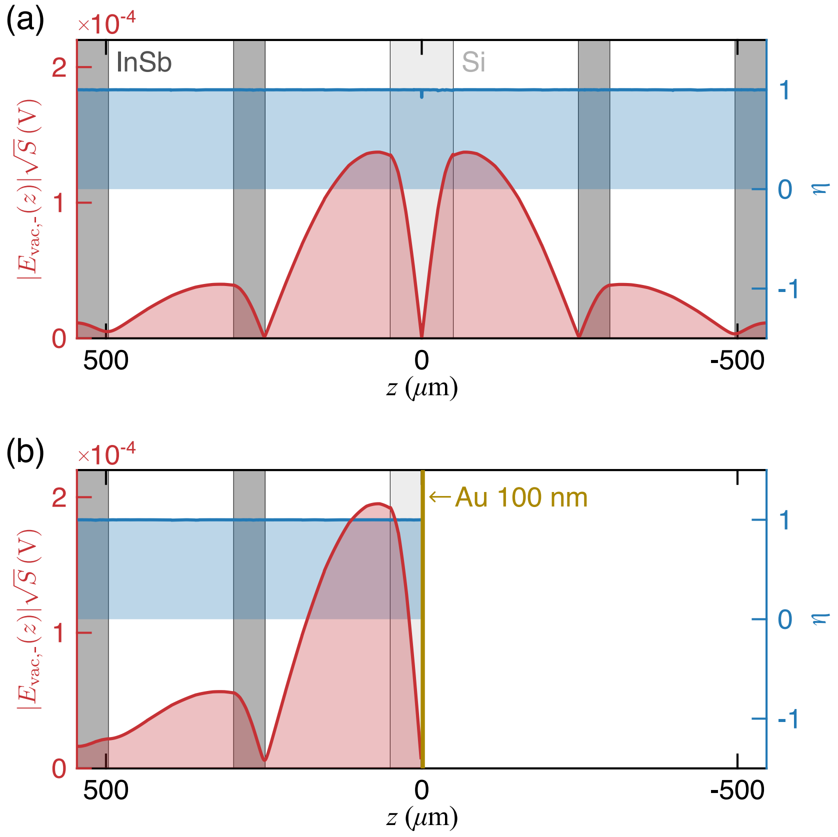

Supplementary Fig. 7 Spatial profiles of the circular component of cavity electric fields in the vacuum state. The vacuum electric field profile, $|E_{\text{vac,-}}(z)|\sqrt{S}$, for (a) the chiral 1D-PCC and (b) the chiral Tamm cavity, where S represents the surface area of the cavity. The light and dark rectangles denote Si and InSb layers, respectively. The yellow line in (b) represents the gold layer.

Supplementary References

[1] Passler, N. C. & Paarmann, A. Generalized 4 × 4 matrix formalism for light propagation in anisotropic stratified media: Study of surface phonon polaritons in polar dielectric heterostructures. *J. Opt. Soc. Am. B* **34**, 2128–2139 (2017).

[2] Meng, F. *et al.* Nonlocal collective ultrastrong interaction of plasmonic metamaterials and photons in a terahertz photonic crystal cavity. *Opt. Express* **27**, 24455–24468 (2019).

[3] Messelot, S., Coeymans, S., Tignon, J., Dhillon, S. & Mangeney, J. High Q and subwavelength THz electric field confinement in ultrastrongly coupled THz resonators. *Photon. Res.* **11**, 1203 (2023).

[4] Wang, Z., Gogna, R. & Deng, H. What is the best planar cavity for maximizing coherent exciton-photon coupling. *Appl. Phys. Lett.* **111**, 061102 (2017).

[5] Tay, F. *et al.* Multimode ultrastrong coupling in three-dimensional photonic-crystal cavities. *Nat. Commun.* **16**, 3603 (2025).
